# Complete Genome Sequence of Avian Paramyxovirus (APMV) Serotype 5 Completes the Analysis of Nine APMV Serotypes and Reveals the Longest APMV Genome

**DOI:** 10.1371/journal.pone.0009269

**Published:** 2010-02-17

**Authors:** Arthur S. Samuel, Anandan Paldurai, Sachin Kumar, Peter L. Collins, Siba K. Samal

**Affiliations:** 1 Virginia-Maryland Regional College of Veterinary Medicine, University of Maryland, College Park, Maryland, United States of America; 2 Laboratory of Infectious Diseases, National Institute of Allergy and Infectious Diseases, Bethesda, Maryland, United States of America; Institut Pasteur, France

## Abstract

**Background:**

Avian paramyxoviruses (APMV) consist of nine known serotypes. The genomes of representatives of all APMV serotypes except APMV type 5 have recently been fully sequenced. Here, we report the complete genome sequence of the APMV-5 prototype strain budgerigar/Kunitachi/74.

**Methodology/Principal Findings:**

APMV-5 Kunitachi virus is unusual in that it lacks a virion hemagglutinin and does not grow in the allantoic cavity of embryonated chicken eggs. However, the virus grew in the amniotic cavity of embryonated chicken eggs and in twelve different established cell lines and two primary cell cultures. The genome is 17,262 nucleotides (nt) long, which is the longest among members of genus *Avulavirus*, and encodes six non-overlapping genes in the order of 3′N-P/V/W-M-F-HN-L-5′ with intergenic regions of 4–57 nt. The genome length follows the ‘rule of six’ and contains a 55-nt leader sequence at the 3′end and a 552 nt trailer sequence at the 5′ end. The phosphoprotein (P) gene contains a conserved RNA editing site and is predicted to encode P, V, and W proteins. The cleavage site of the F protein (G-K-R-K-K-R↓F) conforms to the cleavage site motif of the ubiquitous cellular protease furin. Consistent with this, exogenous protease was not required for virus replication *in vitro*. However, the intracerebral pathogenicity index of APMV-5 strain Kunitachi in one-day-old chicks was found to be zero, indicating that the virus is avirulent for chickens despite the presence of a polybasic F cleavage site.

**Conclusions/Significance:**

Phylogenetic analysis of the sequences of the APVM-5 genome and proteins versus those of the other APMV serotypes showed that APMV-5 is more closely related to APMV-6 than to the other APMVs. Furthermore, these comparisons provided evidence of extensive genome-wide divergence that supports the classification of the APMVs into nine separate serotypes. The structure of the F cleavage site does not appear to be a reliable indicator of virulence among APMV serotypes 2–9. The availability of sequence information for all known APMV serotypes will facilitate studies in epidemiology and vaccinology.

## Introduction

Paramyxoviruses constitute a large family that includes important respiratory and systemic pathogens of humans and animals [1]. Some members of the family are responsible for major diseases, others cause inapparent infections, and the pathogenic potential of others remains unknown. Family *Paramyxoviridae* is divided into two subfamilies: *Paramyxovirinae* and *Pneumovirinae*. Subfamily *Paramyxovirinae* comprises five genera: *Rubulavirus* {including mumps viruses [MuV], human parainfluenza virus types [HPIV 2 and 4], parainfluenza virus 5 [PIV5] (formerly simian virus type 5 [SV5]}, *Respirovirus* {including Sendai viruses [SeV], human parainfluenza virus types 1 and 3 [HPIV 1 and 3]}, *Morbillivirus* {including measles virus [MeV], canine distemper viruses [CDV]}, *Henipavirus* {including Hendra [HeV] and Nipah [NiV] viruses}, and *Avulavirus* {including nine serotypes of avian paramyxoviruses [APMV-1 to -9]}. Subfamily *Pneumovirinae* is divided into two genera: *Pneumovirus* {including human respiratory syncytial virus [HRSV] and bovine respiratory syncytial virus [BRSV]} and *Metapneumovirus* {human metapneumovirus [HMPV] and avian metapneumovirus [AMPV]}[2].

Members of the family *Paramyxoviridae* are pleomorphic, enveloped viruses containing a negative-sense, single-stranded RNA genome. The viral genomes vary in length between 13 to 19 kb and contain 6–10 tandemly linked genes that encode at least 7 and as many as 12 different proteins [1]. For members of subfamily *Paramyxovirinae*, efficient RNA replication requires that the nucleotide (nt) length of the genome is an even multiple of six, known as ‘rule of six’, reflecting the precise packaging of the polynucleotide in the nucleocapsid [3], [4]. The 3′ and 5′ ends of the genome contain short extragenic sequences known as the ‘leader’ and ‘trailer’, regions, respectively. The viral RNA polymerase begins transcription at the 3′ end and proceeds downstream in a sequential manner generating individual mRNAs by a start-stop mechanism guided by gene-start (GS) and gene-end (GE) signals that flank each gene. Non-coding intergenic sequences (IGS) are present between gene boundaries and are not copied into mRNAs. During RNA replication, the GS and GE signals are ignored and a complementary copy of the genome (the antigenome) is synthesized, which serves as the template for synthesis of progeny genome.

All members of family *Paramyxoviridae* encode a nucleoprotein (N), a phosphoprotein (P), a matrix protein (M), a fusion glycoprotein (F), an attachment glycoprotein that in the case of the APMVs is a hemagglutinin-neuraminidase (HN), and a large polymerase protein (L) [1]. The N protein binds the entire length of the viral genomic and antigenomic RNAs to form a stable nucleocapsid that is the template for transcription and RNA replication. The P protein associates with monomeric N protein destined for nucleocapsid assembly and serves as a polymerase co-factor essential for RNA synthesis [5]. The M protein lines the inner membrane of the viral envelope and plays a major role in virion morphogenesis [6], [7]. The F protein mediates viral penetration and syncytium formation. F is synthesized as an inactive precursor F0 that is activated by cleavage by host protease into F1 and F2 subunits. HN initiates infection by binding to sialic acid-containing host-cell surface receptors. HN protein also possesses neuraminidase activity that prevents self-aggregation by progeny viruses. The large protein (L) is the viral RNA dependent RNA polymerase. In addition, most members of subfamily *Paramyxovirinae* encode two additional accessory proteins, V and W (or I, in case of genus *Rubulavirus*) from the P gene by RNA editing during transcription. RNA editing involves the co-transcriptional insertion of one or more non-templated G residues into the nascent P mRNA by the viral polymerase at a conserved RNA editing motif located midway along the P gene. This results in translational frameshifts that access alternative open reading frames downstream, generating the V and W/I proteins. These are proteins whose N-terminal domain is identical to that of P, but each of which have a unique C-terminal domain encoded by the alternative reading frame. The V protein has a conserved cysteine rich zinc finger binding motif in its unique C-terminal domain that is necessary for the activity of V in regulating RNA synthesis and antagonizing the host interferon system [8], [9].

Most of the paramyxoviruses that are isolated from avian hosts are classified in genus *Avulavirus* of subfamily *Paramyxovirinae*: the only exceptions are the avian metapneumoviruses, which are classified in genus *Metapneumovirus* of subfamily *Pneumovirinae* due to substantial differences in genome organization and sequence relatedness [1]. Genus *Avulavirus* is composed of nine recognized serotypes (APMV-1 through APMV-9), a classification that is based on Hemagglutination Inhibition (HI) and Neuraminidase Inhibition (NI) assays [10]. APMV-1 is composed of the many naturally-occurring strains of Newcastle disease virus (NDV) and is the only well-characterized serotype, owing to the high morbidity, mortality, and economic loss caused by highly virulent strains. APMV-2 and -3 also have been reported to cause significant disease in poultry [11], [12], whereas the pathogenic potential of APMV-4 to -9 is generally unknown [13]. Recently, APMV-3 infections in Neophema species of birds was shown to cause 70% mortality [14]. Infections from APMV-4, -8 and -9 appear to be restricted to ducks and geese. APMV-6 and -7 infections in turkeys cause drops in egg production and induce respiratory disease. Currently, our knowledge on the APMV serotypes is expanding with the availability of complete genome sequences of prototypes of all APMV serotypes except APMV-5 [15], [16], [17], [18], [19], [20], [21], [22].

APMV-5 has not been well-characterized but appears to differ from all the other APMV serotypes in two major attributes: (1) the inability to grow in allantoic cavity of embryonated chicken eggs, and, (2) the failure to cause hemagglutination with chicken RBC [23]. APMV-5 was first isolated from an epizootic outbreak involving budgerigars (*Melopsittacus undulatus*) at Kunitachi, Tokyo, Japan in 1974 [23]. This virus was serologically and antigenically distinct from the other previously known APMV serotypes and serves as the prototype virus for this serotype. APVM-5 causes a disease in budgerigars that is characterized by depression, dyspnoea, diarrhea, torticollis and very high mortality. A second outbreak occurred among budgerigars in Brisbane in Queensland, Australia. Experimental infection of APMV-5 isolated from this outbreak caused no signs of disease in young and adult chickens and pigeons, but caused acute fatal enteritis among immature budgerigars [24]. The third known outbreak of APMV-5 disease occurred in budgerigars from UK in 1993 [25]. The clinical signs consisted mainly of vomiting and diarrhea followed by death. Of all the APMVs examined to date, only APMV-1 and APMV-5 have been associated with 100% mortality [23].

As an initial step towards understanding the molecular biology and biological characteristics of APMV-5, we have determined the complete genome sequence of APMV-5 prototype strain APMV-5/budgerigar/Kunitachi/74 (GeneBank accession no. GU206351). An understanding of the molecular and biological characteristics of APMV-5 is important for characterizing the sequence and antigenic relationships within and among the APMV serotypes and for developing vaccines and diagnostic reagents against these viruses.

## Materials and Methods

### 2.1. Virus and Cells

APMV-5 strain budgerigar/Kunitachi/74 was kindly provided by Dr. Ian Brown, the Veterinary Laboratories Agency, Weybridge, Surrey, UK. The virus was propagated in the African green monkey kidney (Vero) cell line. Vero cells were grown in Dulbecco's minimum essential medium (DMEM) containing 10% fetal bovine serum (FBS) and incubated at 37°C under 5% CO_2_. Cell monolayers were infected with a 10^3^ dilution of the original virus stock; after 2h of adsorption, the viral inoculum was replaced with maintenance medium containing 2% FBS. The infected cells were observed daily for cytopathic effects (CPE), which was evident beginning on day 3. The infected cell culture supernatant was examined daily for hemagglutination (HA) activity using chicken RBCs, but this failed to detect virus growth. On day 5 post-infection, when CPE was extensive, the infected cells were scraped into the cell culture medium and pelleted, and the cell pellet was resuspended in a small volume of collected medium and subjected to three cycles of freezing and thawing. This suspension was clarified by low speed centrifugation and the collected medium was pooled and stored at −80°C as virus stock. The virus titer was determined by plaque assay in Vero cells using 0.8% methylcellulose overlay and staining with 1% crystal violet 5 days post-infection. Growth of the virus was evaluated in twelve established cell lines namely, Vero, chicken embryo fibroblast (DF-1), Madin-Darby Canine Kidney (MDCK), human epidermoid carcinoma (HEp-2), Baby Hamster Kidney (BHK-21), Bovine Turbinate (BT), Pig Kidney (PK-15), Quail fibrosarcoma (QT-35), Rabbit Kidney (RK-13), Human cervical carcinoma (HeLa), Madin-Darby Bovine Kidney (MDBK), and duck embryo (CCL-141), all the cell lines were obtained from the American Type Culture Collection (ATCC, Manassas, VA). Growth was also evaluated in two primary cell cultures, namely chicken embryo fibroblast (CEF) and chicken embryo kidney (CEK) cells, which were cultured from 10 and 20-day-old specific pathogen free (SPF) embryonated chicken eggs, respectively, using standard procedures. A total of three serial passages of the virus were made in each cell line to examine virus growth. At each passage level, the cells were collected on day 5-x post-infection, when CPE was apparent and freeze-thawed as described above, and the one-tenth of the collected medium was used to inoculate the next passage. The virus failed to grow in the allantoic cavity of 9-day-old embryonated chicken eggs, but was successfully propagated in the amniotic cavity of 8-day-old embryonated chicken eggs. The infected amniotic fluid was harvested 3 days post-inoculation and the virus was titered using the plaque assay described above. The growth of virus in each cell type was observed with and without the presence of 10% allantoic fluid, or 1–5 µg/ml of acetyl trypsin (Invitrogen, USA), or 1–5 µg/ml of α-chymotrypsin (Sigma), as potential sources of protease for cleavage of the F protein if necessary. All research involving animals have been approved by the members of the institutional animal care and use committee (IACUC).

### 2.2. Virus RNA Isolation and Sequence Analysis

Viral RNA was isolated from infected Vero cells using RNeasy kit according to the manufacturer's instructions (QIAGEN, USA). Reverse transcription (RT) was performed using Superscript II RT (Invitrogen) and an oligo dT primer. The resulting first strand cDNA was PCR-amplified (Recombinant Taq DNA polymerase, Invitrogen) using degenerate consensus primers as the forward primers and either oligo dT or a degenerate consensus primer as the reverse primers. The degenerate consensus forward and reverse primers were designed using published sequences of the viral genomes of members of genera *Avulavirus*, *Rubulavirus*, *Respirovirus* and *Morbillivirus* (accession numbers given below). Specifically, the N, P, M, F, HN and L nucleotide sequences of selected viruses were aligned, and highly-conserved sequence segments were identified and used to design minimally-degenerate primers of 22–24 nt in length. The following degenerate consensus forward primers genes (N = A/C/G/T, S = G/C, M = A/C, W = A/T, K = G/T, R = A/G) were used in conjunction with oligo dT: (i) the N gene forward primer AP-5N380F (5′-NTKCGTCWCTTGCTKCACGAARCA-3′), which would prime at APMV-5 nt 382–405, within the N gene, yielding a 1350 bp length amplicon; (ii) the P gene forward primer AP-5PF 2315 (5′-KCACWCTGCNACASATCAGMRTCC-3′), which would prime at APMV-5 nt 2315–2338, within the P gene, yielding a 1305 bp length amplicon; (iii) the M gene forward primer AP-5MF 4465 (5′-TGGWGTCTACAAARCTCATATCTG-3′), which would prime at APMV-5 nt 4465–4488, within the M gene, yielding a 952 bp length amplicon; (iv) the F gene forward primer AP-5FF 5981 (5′-TTGGTTTGGCAWCTRCTRCACARR-3′), which would prime at APMV-5 nt 5961–5984, within the F gene, yielding a 1505 bp length amplicon; (v) the HN gene forward primer AP-5HNF 7888 (5′- TTAGATARRRTTWCTGTTRAGGTA-3′), which would prime at APMV-5 nt 7888–7911, within the HN gene, yielding a 1824 bp length amplicon; and (vi) the L gene forward primer AP-5LF 14337 (5′- CTRTTTACRTCAGCWGCKCRAGAC-3′), which would prime at APMV-5 nt 14337–14360, within the L gene, yielding a 2368 bp length amplicon. In addition, a second internal L gene forward primer AP-5L1F (5′-CCCTWTKKTCTCTCATWGATACTA-3′), which would prime at APMV-5 nt 10618–10642, within the L gene, was used in conjunction with reverse primer AP-5L1R (5′- WCTTKRTCCTCKCAWTGCTGWTCT-3′), which would prime at APMV-5 nt 13595–13619, within the L gene, yielding a 3002 bp length amplicon. PCR was carried out using a cycling sequence of 95°C for 5 min followed by 35 cycles of 95°C for 1 min, 45°C for 1 min and 72°C for 2 min, which was then followed by a final extension of 72°C for 10 min. New sets of primers were designed from the sequence analysis of these virus-specific cDNAs for determining the remaining sequence. PCR products for determining the sequences of the 3′ and 5′ termini of the genome were made by rapid amplification of cDNA ends (3′ and 5′ RACE, respectively) from the viral RNA using manufacturer's protocol (Invitrogen) with modification from Subbiah et al., 2008. Briefly purified viral RNA from infected cells was ligated using T4 RNA ligase to a 5′-phosphorylated and 3′ blocked RNA oligonucleotide adapter (5′-CCAAAACGCCAUUUCCACCUUCUCUUC-3′). The ligated RNA was subjected to RT using adapter 2 (5′-GAAGAGAAGGTGGAAATGGCGTTTTGG-3′) as the primer. The cDNA was PCR amplified using adapter 2 as the forward primer and a gene specific reverse primer obtained from the N gene (5′-GTTTAATCTTAAGAGCCCTCCTGTC-3′), which would prime at APMV-5 nt 200–224, within the N gene. For determining the 5′ end of the genome, a 5′RACE kit (Invitrogen) was utilized. Purified viral RNA was reverse transcribed using an L gene specific primer (5′-CTAACGCGCCTTCTTACATCCGACGTTCAT-3′), which would prime at APMV-5 nt 16423–16452, within the L gene. The resulting cDNA was subjected to a tailing reaction with dCTP using terminal deoxynucleotidyl transferase, and then was PCR amplified using same L gene specific primer and an anchored oligo dG primer. PCR products were gel purified and were sequenced directly or were cloned into the TOPO TA cloning vector (Invitrogen) and positive clones were sequenced using M13 forward and M13 reverse primers. DNA sequencing was performed in a 3130*xl* genetic analyzer (Applied Biosystems Inc, USA) according to the manufacturer's instruction. The entire genome sequence was determined at least three times: once by analysis of cloned cDNAs and twice by direct sequencing of uncloned RT-PCR products, providing a consensus sequence.

### 2.3. Sequence and Phylogenetic Tree Analysis

Sequence analysis, BLAST searches, and prediction of ORFs were carried out using the SeqMan and EditSeq programs, and PCR primers were designed using the PrimerSelect program in DNASTAR Lasergene 8 (software suite for sequence analysis, version 8.0.2(13) 412). Bootstrap values in the phylogenetic trees were calculated using 1000 replicas. Construction of phylogenetic trees and divergence analysis was performed by maximum parsimony method using MEGA 4 software (Molecular Evolutionary Genetics Analysis) [26].

### 2.4. Database Accession Numbers

The complete genome sequence of APMV-5 strain budgerigar/Kunitachi/74 was submitted to GenBank (accession number GU206351). Accession numbers for other paramyxovirus sequences were as follows. Avulaviruses: APMV-1, AF077761; APMV-2, EU338414; APMV-3, EU403085; APMV-4KR, EU877976; APMV-4HK, FJ177514; APMV-6TW, NC 003043; APMV-6HK, EU622637; APMV-6FE, EF569970; APMV-7, FJ231524; APMV-8DEL, FJ215863; APMV-8WAK, FJ215864; APMV-9, EU910942. Rubulaviruses: hPIV-2 NC_003443; PIV5 (also known as SV-5) NC_006430; MuV NC_002200; simian virus 41 (SV41) NC_006428. Respiroviruses: hPIV-1, NC_003461; hPIV-3, NC_001796; SeV, NC_001552, BPIV-3, NC_002161. Henipaviruses: NiV, NC_002728; HeV, NC_001906. Morbilliviruses: CDV, NC_001921; MeV, AF266288; phocine distemper virus (PDV), NC_006383; rinderpest virus (RPV), NC_006296; peste des petits ruminants virus (PPRV), NC_006383; dolphin morbillivirus (DMV), NC_005283; other paramyxovirus: Atlantic salmon paramyxovirus (ASPV), EF646380; Beilong virus (BeV), NC_007803; Fer-de-Lance virus (FDLV), NC_005084; J virus (JV), NC_007454; Menangle virus (MenV), NC_007620; Mossman (MoV), NC_005339; Tupaia paramyxovirus (TpV), NC_002199; Pneumoviruses: HRSV, NC001781; BRSV, NC001989. Metapneumoviruses: AMPV, NC007652; HMPV, NC004148.

## Results

### 3.1. Growth Characteristics of APMV-5

APMV-5 strain Kunitachi produced CPE in Vero cells, resulting in cell rounding and detachment as well as the formation of large syncytia observed 3 days post-infection. The virus produced distinct plaques in Vero cells under methylcellulose overlay. Supplementation with 10% fresh allantoic fluid, acetyl trypsin (1–5 µg/ml), or α-chymotrypsin (1–5 µg/ml) did not enhance the growth of the virus, indicating the lack of a requirement for external protease for efficient cleavage of the F protein. Consistent with previous reports [16], [18], [20], APMV-5 did not cause hemagglutination of chicken, turkey, guinea pig, horse or human-O-group RBCs. We note that weak hemagglutinin (HA) activity (up to 2^4^ HAU) was observed when we purified and concentrated the APMV-5 strain Kunitachi virus (not shown). However, we observed a similar level of weak HA activity with other purified preparations of viruses that are known to lack HA activity and were analyzed in parallel as negative controls, namely blue tongue virus, infectious bovine rhinotracheitis, and infectious bronchitis virus, and even uninfected Vero cell lysates gave a titer of up to 2^2^ HAU. Thus, we concluded that this weak HA activity was a non-specific artifact of using highly concentrated virus. These results identified strain Kunitachi as the only APMV analyzed to date that appears to lack a typical virion HA activity. Twelve different cell lines from mammalian and avian species (BHK-21 HEp-2, Vero, HeLa, MDCK, DF-1, MDBK, PK-15, RK-13, CCL-141, QT-35 and BT) and two primary chicken cell cultures (CEF and CEK) were evaluated to identify cell substrates that can support the replication of APMV-5. The virus was subjected to three passages in each cell line, and the peak titer was determined by plaque assay. The virus grew most efficiently in chicken DF-1 cells, followed by Vero and BHK-21 cells. The peak titers (PFU/ml) of the different cell lines tested were: Vero, 6.5×10^7^; BHK-21, 4.6×10^5^; DF-1, 4.4×10^10^; HeLa, 3×10^5^; MDCK, 2.8×10^5^; MDBK, 3×10^3^; HEp-2, 1.1×10^3^; QT-35, 2×10^2^; CEK, 1.3×10^3^; and CEF, 2.5×10^3^. Virus growth was not detectable in PK-15, RK-13, BT and CCL-141 cells using plaque assay but RT-PCR analysis of the infected cell culture supernatant and the cell pellet indicated a low level of virus replication in each of these cell cultures. Interestingly, APMV-5 failed to grow in the allantoic cavity of 9-day-old embryonated chicken eggs, which is contrary to all other APMV serotypes. But APMV-5 grew in the amniotic cavity of 8-day-old embryonated chicken eggs, yielding a titer of 1.2×10^3^ PFU/ml. The virus did not cause death of the chicken embryos in a mean death time (MDT) assay even after 168 h post infection. The intracerebral pathogenicity index (ICPI) in one-day-old chicks for APMV-5 strain Kunitachi was zero, indicating the virus is non pathogenic for chickens.

### 3.2. Determination of the Complete Genome Sequence of APMV-5

We determined a complete consensus sequence of the genome of APMV-5 strain Kunitachi (GenBank accession no. GU206351). RT-PCR products spanning much of the genome were prepared using degenerate PCR primers derived from consensus sequences identified by sequence alignment of multiple members of the *Avulavirus*, *Rubulavirus*, *Respirovirus*, and *Morbillivirus* genera (see Materials and Methods). Based on the sequences of these initial cDNAs, additional primers were designed to complete the analysis. The entire sequence was confirmed in uncloned PCR products, providing a consensus sequence.

**Figure 1:**
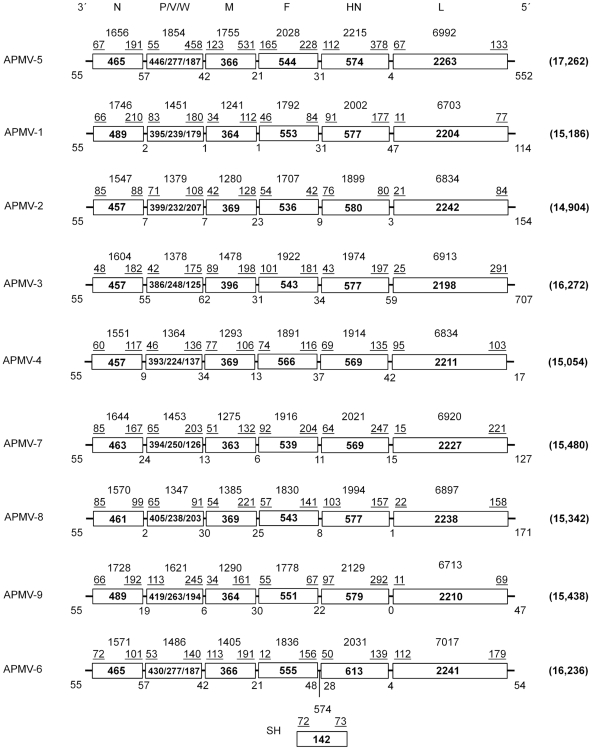
Gene map of APMV-5 and comparison with prototype strains for the other eight APMV serotypes (maps not to scale). Individual genes are indicated by rectangles, with the gene names given at the top of the Figure. The amino acid length(s) of the encoded protein(s) is shown in each box. The nt length of the gene is shown over each box. The lengths of the non-translated upstream and downstream regions (which also are represented in the total gene length) are indicated by underlined values immediately over each box. The nt lengths of the non-coding leader, intergenic, and trailer regions are shown under each map, and the total genome nt length is shown in parentheses to the right. The SH gene is present in APMV-6 but not in the other APMVs. Upstream UTRs include the GS sequence and the downstream UTRs include the GE sequence. The prototypes strains used here and in the other Figures and Tables are as follows: APMV-5, APMV-5/budgerigar/Kunitachi/74 (present study); APMV-1, Newcastle disease virus (strain LaSota/46); APMV-2, APMV-2/Chicken/California/Yucaipa/56; APMV-3, APMV-3/PKT/Netherland/449/75; APMV-4, APMV-4/duck/Hongkong/D3/75; APMV-6, APMV-6/duck/HongKong/18/199/77; APMV-7, APMV-7/dove/Tennessee/4/75; APMV-8, APMV-8/goose/Delaware/1053/76; APMV-9, APMV-9/duck/New York/22/1978.

The genome organization of APMV-5 strain Kunitachi is 3′-leader-N-P/V/W-M-F-HN-L-trailer-5′ (Fig 1), similar to the organization of the other members of genus *Avulavirus* except for APMV-6, which has an additional small hydrophobic (SH) protein gene between the F and HN genes [19]. Thus, each of the APMV serotypes contains six genes except for APMV-6, which has seven. Major features of the genome of APMV-5 strain Kunitachi are summarized in Fig. 1 and are compared with prototype members of the other APMV serotypes. The APMV-5 genome consists of 17,262 nt and thus is the longest among the APMVs. The genome nt length is a multiple of six, conforming to the “rule of six”, as is the case for all members of subfamily *Paramyxovirinae* analyzed to date [4], [27].

Although the genome length of APMV-5 is substantially greater than for the other serotypes, this was not reflected in increased lengths for the encoded proteins: the amino acid lengths predicted for the APMV-5 proteins are very similar to those of the cognate proteins in the other APMV serotypes (Fig. 1). Instead, the increased genome length of APMV-5 was mostly due to the presence of unusually long 3′(downstream) untranslated regions (UTR) in the P (458 nt), M (531 nt), and HN (378 nt) genes, as well as the presence of a long trailer region (below). The UTRs of all the APMVs sequenced to date vary greatly in length. The length of 5′ (upstream) UTRs of the APMV-5 N, P, M, F, HN and L genes are 67, 55, 123, 165, 112 and 67, nt, respectively (Figure 1). For comparison, the range of nt lengths of the 5′ UTRs of the N, P, M, F, HN and L genes of the other eight APMV serotypes are 48–85, 42–113, 34–113, 12–101, 43–103, and 11–112, respectively. The lengths of the 3′ UTRs of the APMV-5 N, P, M, F, HN and L genes are 191, 458, 531, 228, 378 and 133, respectively. For comparison, the nt lengths of the 3′ UTRs of the N, P, M, F, HN and L genes of the eight other APMV serotypes range between (88–210), (91–245), (106–221), (42–204), (80–292) and (69–291), respectively. Consistent with the longer lengths of some of the UTRs of APMV-5, the percentage of the APMV-5 genome that encodes proteins is 81%, which is less than average coding percentage (92%) of other members of subfamily *Paramyxovirinae* [1], [28].

The genome of APMV-5 has 44%, 48%, 42%, 42%, 55%, 48%, 48% and 44% nt sequence identity with the genomes of APMV-1, -2, -3, -4, -6, -7, -8 and -9, respectively (Table 1). The GC content of the genome of APMV-5 is 40%, which is similar to APMV-7 (39%) but significantly lower compared to the GC contents of the genomes of APMV-1 (46%), APMV-2 (47%), APMV-3 (46%), APMV-4 (47%), APMV-6 (46%), APMV-8 (43%) and APMV-9 (45%).

**Table 1:** Percent nucleotide sequence identities between the complete genomes of prototype strains of the nine APMV serotypes.

	APMV-2	APMV-3	APMV-4	APMV-5	APMV-6	APMV-7	APMV-8	APMV-9
APMV-1	46	44	43	44	45	45	46	60
APMV-2		43	44	48	50	51	60	45
APMV-3			46	42	44	43	44	43
APMV-4				42	44	43	43	43
APMV-5					55	48	48	44
APMV-6						49	49	45
APMV-7							51	45
APMV-8								46

**Figure 2:**
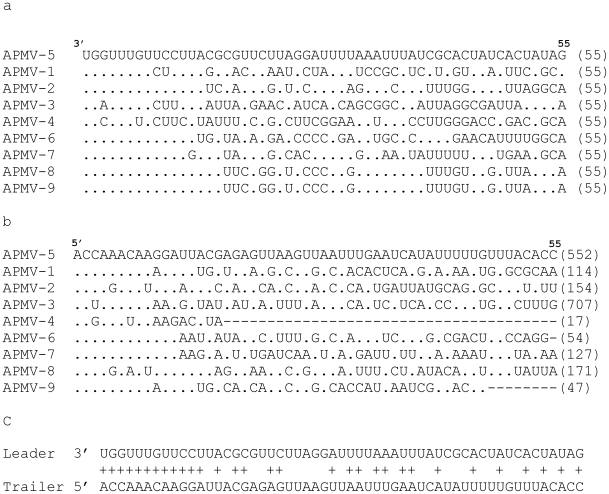
Alignment and comparison of Leader and trailer sequences of APMV-5 with other APMV prototype strains. Sequence alignments (negative-sense) of the (a) 3′-leader (b) 5′-trailer regions of APMV-5 with those of prototypes strains of the other eight APMV serotypes, and (c) terminal complementarity between the leader and trailer of APMV-5. Dots in (a) and (b) indicate identical nucleotides to APMV-5; crosses in (c) indicate complementarity. Gaps are indicated by dashes. The numbers in the parenthesis indicate total number of nucleotides.

The 3′-leader sequence of APMV-5 consists of 55 nt, a length that is conserved among all of the APMV and among almost all of the members of the subfamily *Paramyxovirinae*. The APMV-5 leader has a high degree of nucleotide sequence identity with those of the other APMVs (Fig. 2a). The sequence of the first 12 nt of the leader region of APMV-5 (3′-UGGUUUGUUCCU-5′, negative-sense) is identical to that of APMV-2, -6, -7, -8 and -9. The first 8 nt of the leader sequence are the same for all the members of genus *Avulavirus* sequenced to date except for a 1-nt change in the third position of APMV-3, where residue G is replaced with residue A, and 2 nt changes at the third and seventh positions of APMV-4, involving G to C and G to U changes, respectively.

The length of the 5′-trailer region of APMV-5 is 552 nt, which is second longest next to APMV-3 (707 nt), whereas the shortest trailer region among the APMV serotypes is 17 nt, for APMV-4. The length of the trailer region is variable among the members of the subfamily *Paramyxovirinae*. The sequence of the last 12 nt of the APMV-5 trailer sequence is identical to those of APMV-6 and -7, and 11 nt out of the last 12 nt are identical to those of APMV-1 and -9 (Fig. 2b). The first 12 nt of the leader and trailer sequences of APMV-5 are 100% complementary to each other, which is suggestive of conserved promoter elements at the 3′ termini of the genome and antigenome (Fig. 2c).

### 3.3. Sequences of the GS and GE Motifs and the IGS

The GS sequences of the various genes of APMV-5 are identical at positions 1, 3, 4 and 5, with a consensus sequence (negative-sense) of 3′-CCCCCUUNNN-5′ (Table 2). The GE sequences are identical at positions 1–3 and 6–10, with a consensus (negative-sense) of 3′-AAU(A/U)NU_5_-5′(Table 2). Interestingly, the GS and GE signals of the APMVs sequenced to date have a high degree of identity, implying that polymerase recognition of these cis-acting sequences is conserved within the genus (Table 3). The hexamer phasing positions of the GS signals of APMV-5 are 2,5,2,2,3 and 2 which are different from those of APMV-1 (2,4,3,3,2 and 5), -2 (2,2,2,3,3 and 3), -3 (2,5,5,2,2 and 1), -4 (2,2,2,6,2 and 2), -6 (2,2,2,2,2,4 and 4), -7 (2,2,4,1,2 and 4), -8 (2,2,2,2,4 and 1) -9 (2,3,4,4,4 and 3). Thus, the hexamer phasing positions of the GS signals in the APMVs sequenced to date are not conserved other than that of the N gene being 2.

**Table 2:** The gene-start and gene-end sequences of APMV-5 strain Kunitachi.

Gene-start†	Gene	Gene-end†
CUCCCUCUUA	N	AAUAUUUUUU
CCCCCUUGGU	P	AAUUAUUUUUUU
CCCCCUUGAC	M	AAUAUUUUUU
CCCCCUUACU	F	AAUAUUUUUU
CCCCCUUCUA	HN	AAUUCUUUUUUU
CCCCCCUUUU	L	AAUUCUUUUUU
CCCCCUUNNN *		AAU(A/U)NU_5–7_ *

† Gene-start and gene-end sequences are given in negative sense.

* Consensus sequence.

**Table 3:** The consensus sequences of the gene-start and gene-end signals of prototypes strains of the nine APMV serotypes‡.

APMV	Gene-start	Gene-end
APMV-5	CCCCCUUNNN	AAU(A/U)NU_5–7_
APMV-1	UGCCCAUCUU	AAU(C/U)_2_U_6_
APMV-2	CCCCCGCUGU	AAUUAU_6_
APMV-3	UCCUCGCCUU	AAUUAU_6_
APMV-4	CACCCCUUCC	AAUUAAU_5_
APMV-6	CUC_5–6_UUC	AAU(N_1–2_)AU_4–6_
APMV-7	CUCCCNCUNN	AAUNNUUUNU_1–3_
APMV-8	CCCCCGC(U/G)N	AAUUNU_6_
APMV-9	UGCCCAUCUU	AAUNU_6_

‡ Gene-start and gene-end sequences are given in negative sense.

The IGS of APMV-5 differed in sequence and ranged in length from 4 to 57 nt. The IGS between the N/P, P/M, M/F, F/HN and HN/L genes are 57, 42, 21, 31 and 4 nt, respectively. In comparison, those of the other APMVs have a similar size range and are between 1–47 nt for APMV-1, 3–23 nt for APMV-2, 31–62 nt for APMV-3, 9–42 nt for APMV-4, 4–57 nt for APMV-6, 6–24 for APMV-7, 1–30 for APMV-8 and 0–30 for APMV-9 (Figure 1).

### 3.4. The Nucleoprotein (N) Gene and the N Protein

The N gene of APMV-5 is 1656 nt long and encodes an N protein of 465 amino acids (aa) with a predicted molecular weight (MW) of 51,172 and isoelectric point (p*I*) of 6.07. The N protein contain a highly conserved motif, 324-FAPANYTLLYSYAMG-338 (F-X4-Y-X3-Φ-S-Φ-A-M-G, where, X is any amino acid and Φ is any aromatic amino acid) that has been identified in other members of the subfamily *Paramyxovirinae* and is thought to be responsible for N–N self-assembly during genomic RNA binding [29]. The amino acids at position 325, 326 and 333 in the motif are alanine (A), proline (P) and tyrosine (Y), respectively, which are conserved in all known APMVs (Table 4). The N protein of APMV-5 has an aa sequence identity of 38%, 52%, 37%, 37%, 62%, 52% 55% and 37% with the N proteins of APMV-1, -2, -3, -4, -6, -7, -8 and -9, respectively (Table 5). The APMV-5 N protein has an aa sequence identity of 33%, 20%, 29%, 29%, and 11%, with the N proteins of MuV (*Rubulavirus*), SeV (*Respirovirus*), MeV (*Morbillivirus*), NiV (*Henipavirus*), and AMPV (*Metapneumovirus*), respectively.

**Table 4:** Comparison of amino acid sequence motifs observed in the N, M, HN and L proteins of prototype strains of the nine APMV serotypes.

APMV	N self-assembly	M late domain	HN sialic acid binding	L domain III	ATP binding motif domain VI
	FXXXXYXXXΦSΦAMG	αPΦΦ	NRKSCS	QGDNQ	KX_21_GXGXG
**APMV-5**	**^324^FAPGNYPLIYSYAMG^338^**	**^22^FPIV^25^**	**^234^NRKSCS^239^**	**^800^QGDNQ^804^**	**^995^KX_21_ GEGSW^1017^**
APMV-1	^322^....E.AQ...F...^336^	^23^....^26^	^234^......^239^	^749^.....^753^	^1756^. . A....^1782^
APMV-2	^324^.....FST.......^338^	^22^Y.L.^25^	^235^......^240^	^773^.....^777^	^1796^. . .....^1822^
APMV-3	^322^......S........^336^	^23^..L.^26^	^237^......^242^	^744^.....^748^	^1752^R . .....^1775^
APMV-4	^322^.....F.HM......^336^	^22^..L.^25^	^229^......^234^	^756^.....^760^	^1767^R . ...Y.^1793^
APMV-6	^324^........M......^338^	^22^...I^25^	^240^......^245^	^776^.....^780^	^1797^. . A.S.G^1823^
APMV-7	^324^...A..T........^338^	^22^...I^25^	^224^......^229^	^767^.....^771^	^1235^. . ..TDQ^1260^
APMV-8	^324^......STM......^338^	^22^..L.^25^	^236^......^241^	^773^.....^777^	^1796^. . .....^1822^
APMV-9	^322^....E.AQ.......^336^	^23^....^26^	^234^......^239^	^749^.....^753^	^1759^. . A....^1785^

**Table 5:** Percent amino acid sequence identities between the proteins of APMV-5 strain Kunitachi and those of prototype strains of the other APMV serotypes.

APMV	Amino acid sequence identity (%)					
	N	P	M	F	HN	L
**APMV-1**	38	20	33	41	35	38
**APMV-2**	52	26	43	47	42	42
**APMV-3**	37	19	31	31	33	35
**APMV-4**	37	22	28	33	30	33
**APMV-6**	62	28	55	55	56	50
**APMV-7**	52	22	45	37	43	44
**APMV-8**	55	27	42	46	41	43
**APMV-9**	37	21	29	37	31	38

### 3.5. The Phosphoprotein (P) Gene and the P, V, and W Proteins

The P gene of APMV-5 is 1854 nt long and encodes a P protein of 446 aa with a MW of 48,906 and p*I* of 5.92. The predicted P protein contains 38 potential sites for phosphorylation, as predicted by the NetPhos 2.0 program of Expasy proteomics server. These include 29 potential sites of serine phosphorylation (28S, 31S, 65S, 67S, 80S, 93S, 98S, 112S, 131S, 138S, 159S, 164S, 171S, 194S, 233S, 240S, 280S, 288S, 301S, 308S, 349S, 379S, 383S, 388S, 410S, 415S, 425S, 427S and 431S), 7 potential sites of threonine phosphorylation (40T, 133T, 156T, 185T, 198T, 222T and 419T), and 2 potential sites for tyrosine phosphorylation (36Y and 193Y). The P protein of APMV-5 has an aa sequence identity of 20%, 26%, 19%, 22%, 28%, 22%, 27% and 21% with the P proteins of APMV-1, -2, -3, -4, -6, -7, -8 and -9, respectively (Table 5). The P protein of APMV-5 has an aa sequence identity of 16%, 12%, 11%, 10%, and 9%, with the P proteins of MuV (*Rubulavirus*), SeV (*Respirovirus*), MeV (*Morbillivirus*), NiV (*Henipavirus*), and AMPV (*Metapneumovirus*), respectively.

**Figure 3:**
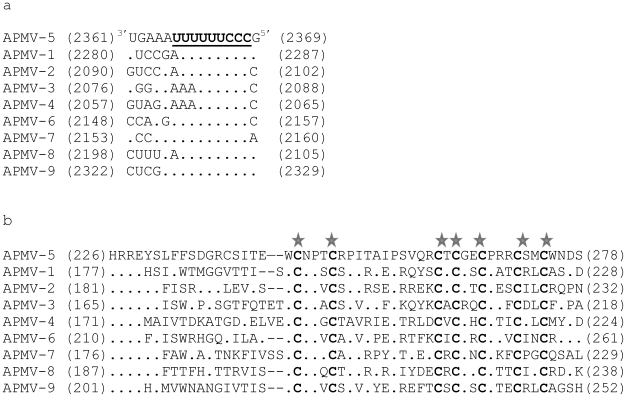
Sequence alignments of P gene editing site and C-terminal region of V protein of all APMV prototype strains. Sequence alignments of (a) the RNA editing site in the P gene (shown in negative-sense) and (b) the C-terminal domain of the V protein of APMV-5 versus prototype viruses of the other eight APMV serotypes. The editing site is underlined. Dots indicate nucleotides identical to those of APMV-5. The conserved cysteine residues in V protein are bolded and marked by stars. The numbers in the parenthesis indicate the corresponding nucleotide position in the complete genome (a) and amino acid position of V gene (b).

The P gene contains a putative editing site 3′-UUUUUUCCC-5′ (negative-sense) at nt positions 537–546 in the P gene (that corresponds to nt positions 2361–2369 in the genome). The sequence of the APMV-5 editing site is exactly same as that in APMV-7 and is similar to those in the other APMV serotypes (Fig. 3a). The addition of a single G residue at the APMV-5 editing site would produce a V mRNA encoding a V protein of 277 aa, with a predicted MW of 31,180. The N-terminal 182 aa domain of the V protein is identical to the corresponding domain in the P protein and is followed by a V-specific C-terminal domain of 95 aa. This V-specific domain contains seven cysteine residues that are exactly conserved in number and spacing in other members of the subfamily *Paramyxovirinae* (Fig. 3b). The V protein of APMV-5 has an aa sequence identity of 25%, 28%, 19%, 26%, 30%, 27%, 30% and 23% with the V proteins of APMV-1, -2, -3, -4, -6, -7, -8 and -9, respectively. The addition of two G residues at the editing site would produce a W mRNA encoding a W protein of 187 aa, with a predicted MW of 20,727. The putative W protein contains an N-terminal domain of 182 aa that is identical to that of P followed by five additional residues that are unique to W. The W protein of APMV-5 has an aa sequence identity of 16%, 22%, 15%, 28%, 28%, 22%, 22% and 24% with the W proteins of APMV-1, -2, -3, -4, -6, -7, -8 and -9, respectively.

### 3.6. The Matrix (M) Gene and the M Protein

The M gene of APMV-5 is 1755 nt long and encodes a M protein of 366 aa with a predicted MW of 39,999. The p*I* value of the M protein is 9.31: this basic nature is thought to mediate ionic interactions with the acidic N protein [1]. The M proteins of some members of the subfamily *Paramyxovirinae* contains a motif (242-**KK**TN**RK**GAD**R**SVLQI**K**E**K**V**RK**-262 in APMV-5) that consists of a bipartite clustering of basic residues. This motif is thought to serve as a nuclear localization signal (NLS) for the protein [6], [30], although the significance of this for infection remains unclear. A protein–protein interaction domain called the ‘late domain’, which contains the motif FPIV and is involved in assembly and budding of viruses, was first identified in the paramyxovirus SV5 [31]. The M protein of APMV-5 contains a potential late domain motif FPIV at aa positions 22–25. Similar motifs are present in the M proteins of other APMV, namely: 22-YPLI-25, APMV-2; 23-FPLI-26, APMV-3; 22-FPLI-25, APMV-4; 22-FPLV-25, APMV-6; 22-FPII-25, APMV-7; 22-FPLV-25, APMV-8; and 23-FPIV-26, APMV-9 (Table 5). In the late domain motif, the amino acid proline (P) in the second position is conserved in all the members of genera *Avulavirus* and *Rubulavirus*, with the consensus pattern α-P-Φ-Φ (α represents an aliphatic amino acid and Φ represents an aromatic amino acid). However, this motif is not observed in the M proteins of members of genera *Respirovirus*, *Morbillivirus* and *Henipavirus* of the subfamily *Paramyxovirinae*. The M protein of APMV-5 has an aa sequence identity of 33%, 43%, 31%, 28%, 55%, 45%, 42% and 29% with the M proteins of APMV-1, -2, -3, -4, -6, -7, -8 and -9, respectively (Table 5). The APMV-5 M protein has an aa sequence identity of 28%, 22%, 20%, 21%, and 14%, with the M proteins of MuV (*Rubulavirus*), SeV (*Respirovirus*), MeV (*Morbillivirus*), NiV (*Henipavirus*), and AMPV (*Metapneumovirus*), respectively.

### 3.7. The Fusion (F) Gene and the F Protein

The F gene of APMV-5 is 2028 nt long and encodes a F protein of 544 aa with a predicted MW of 58,724 and p*I* of 8.34. The F protein is predicted to be a type I transmembrane protein similar to the F proteins of the other members of the family *Paramyxoviridae*. It contains a signal sequence of 25 aa (positions 1–25) at the N-terminal end and a predicted transmembrane domain located at aa sequence positions 494–516. The putative cleavage site of the APMV-5 F_0_ protein precursor is G-**K-R-K-K-R**↓F (aa positions 104–110), with the residue F representing the newly-created N-terminus of the F1 fusion peptide. The cleavage site sequence of APMV-5 conforms to the favored sequence motif for cleavage by the intracellular protease furin, R-X-K/R-R↓F [32]. Interestingly, the cleavage site of APMV-5 contains five tandem lysine and arginine residues (KRKKR), which is the greatest number of basic residues for any of the APMV (Table 6) except for some strains of APMV-1 [33]. The F1 polypeptide of APMV-5 contains heptad repeats at aa positions 134–182 and 455–485, as predicted by the LearnCoil-VMF program, which correspond to the characteristic HRA and HRB heptad repeats that are found in paramyxovirus F proteins and are thought to be important for the conformational changes that lead to membrane fusion [34]. The APMV-5 F protein contains five potential N-linked glycosylation sites located at aa position 74 of the F2 subunit and at aa positions 189, 359, 440 and 464 of the F1 subunit, as predicted by the NetNGlyc 1.0 program of the Expasy proteomics server. The F protein of APMV-5 has an aa sequence identity of 41%, 47%, 31%, 33%, 55%, 37%, 46% and 37% with the F proteins of APMV-1, -2, -3, -4, -6, -7, -8 and -9, respectively (Table 5). The APMV-5 F protein has an aa sequence identity of 33%, 24%, 27%, 24%, and 19%, with the F proteins of MuV (*Rubulavirus*), SeV (*Respirovirus*), MeV (*Morbillivirus*), NiV (*Henipavirus*) and AMPV (*Metapneumovirus*), respectively.

**Table 6:** Comparison of the putative cleavage sites of the F_0_ proteins of prototypes strains of the nine APMV serotypes, and requirements for exogenous protease.

APMV	F_0_ protein cleavage site†	Requirement for exogenous protease‡
**APMV-5**	G**KRKKR**↓FVG	−
APMV-1(Avirulent)	GG**R**QG**R**↓LIG	+
APMV-1(Virulent)	G**RR**Q**KR**↓FIG	−
APMV-2	D**K**PAS**R**↓FVG	−
APMV-3	A**R**P**R**G**R**↓LFG	+
APMV-4	ADIQP**R**↓FIG	−
APMV-6	PAPEP**R**↓LIG	−
APMV-7	TLPSS**R**↓FAG	−
APMV-8	TYPQT**R**↓LIG	+
APMV-9	**R**I**R**EG**R**↓IFG	+

† Basic amino acids (R/K) are underlined and in bold, and the downward arrow indicates the predicted site of cleavage.

‡ Virus replication in cell culture requires the supplementation of 10% v/v fresh allantoic fluid or acetylated trypsin (1 µg/ml).

Note: APMV-1 (Avirulent) denotes strain LaSota, APMV-1 (Virulent) denotes strain GB Texas, APMV-2 to APMV-9 includes all the prototype strains.

### 3.8. The Hemagglutinin–Neuraminidase (HN) Gene and the HN Protein

The HN gene of APMV-5 is 2215 nt long and encodes a HN protein of 574 aa with a predicted MW of 63,911 and p*I* of 6.78. The HN protein is predicted to be a type II integral membrane protein and has a predicted hydrophobic signal/anchor domain located at aa residues 21 and 43 at the N-terminus. There are six potential N-linked glycosylation sites predicted at aa positions 58, 119, 148, 278, 346 and 383. The HN protein contains the unique motif N-R-K-S-C-S at aa positions 234–239, which is thought to be involved in sialic acid binding at the cell surface [35], [36]. This motif is conserved in the HN proteins of all APMVS sequenced to date (Table 4). Comparing the seven conserved residues of the proposed neuraminidase active site found in the HN protein of NDV [37], [38], the corresponding residues in the APMV-5 HN are: R(174), S(202), E(399), R(414), R(504), Y(532) and E(550). In addition, the APMV-5 HN protein has eleven conserved cysteine residues at aa positions 186, 196, 238, 247, 251, 344, 461, 467, 471, 534 and 545. The HN protein of APMV-5 has an aa sequence identity of 35%, 42%, 33%, 30%, 56%, 43%, 41% and 31% with the HN proteins of APMV-1, -2, -3, -4, -6, -7, -8 and -9, respectively (Table 5). APMV-5 HN protein has an aa sequence identity of 33%, 24%, 11%, 16%, and 8%, with the HN proteins of MuV (*Rubulavirus*), SeV (*Respirovirus*), MeV (*Morbillivirus*), NiV (*Henipavirus*) and AMPV (*Metapneumovirus*), respectively.

### 3.9. The Large Polymerase (L) Gene and the L Protein

The L gene of APMV-5 is 6992 nt long and encodes a L protein of 2263 aa with a predicted MW of 255,938 and p*I* of 7.49. The sequence alignment of the APMV-5 L protein showed six linear conserved domains, as found in other members of the family *Paramyxoviridae* (data not shown) [39]. The previously conserved QGDNQ sequence motif in domain III, which is widely conserved and is thought to be involved in L protein transcriptional activity [40], [41], also was present in the L protein of APMV-5 (aa sequence positions 800–804, Table 4). Domain IV of the APMV-5 L protein contains the ATP-binding motif K-X21-G-E-G-S-W which is exactly conserved in APMV-2 and -8, but has one or more amino acid differences compared to the other APMV serotypes (Table 4). The L protein of APMV-5 has an aa sequence identity of 38%, 42%, 35%, 33%, 50%, 44%, 43%, and 38% with the L proteins of APMV-1, -2, -3, -4, -6, -8, and -9, respectively (Table 5). The APMV-5 L protein has aa sequence identity of 34%, 25%, 26%, 25%, and 16%, with the L proteins of MuV (*Rubulavirus*), SeV (*Respirovirus*), MeV (*Morbillivirus*), NiV (*Henipavirus*), and AMPV (*Metapneumovirus*), respectively.

### 3.10. Phylogenetic Tree Analyses

**Figure 4:**
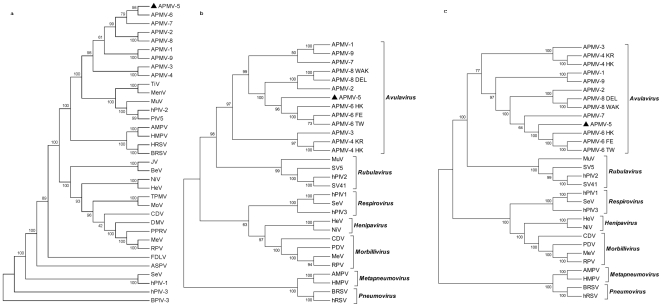
Phylogenetic tree of representative members of family *Paramyxoviridae*. Phylogenic analysis of the complete genome (a) and respective, F (b) and L (c) proteins of members of family *Paramyxoviridae*. The numbers represents the bootstrap values among different viruses. The phylogenetic trees were analyzed by average distance using MEGA 4.1, Molecular Evolutionary Genetics Analysis software.

A phylogenetic tree was constructed from a sequence alignment of the complete genome nt sequences of the prototype members of the nine APMV serotypes (Fig. 4a). In addition, phylogenetic trees were prepared for the N, P, M, F, HN and L proteins of APMV-5 versus the cognate proteins of members of the other eight APMV serotypes and representative viruses of the other four genera of subfamily *Paramyxovirinae* (trees for the F and L protein are shown in Fig 4b and c, respectively). The phylogenetic trees clearly indicated that the APMV-5 was most closely related to the known APMVs, consistent with its inclusion in the genus *Avulavirus*. Furthermore, the phylogenetic analysis of the complete genome sequence and the sequences of the six proteins revealed a closer genetic relatedness of APMV-5 to APMV-6 than to the other APMV serotypes within the genus *Avulavirus*.

## Discussion

The APMVs are frequently isolated from a wide variety of avian species and are grouped into nine serological types based on antigenic tests involving the HN protein. Until recently, very little was known about the molecular details of these common viruses, except for NDV (APMV-1). However, recent sequence analysis of prototypes of APMV-2, -3, -4, -6, -7, -8 and -9 has revealed many of the molecular details of these viruses [16], [17], [18], [19], [20], [21], [22]. Prior to this report, APMV-5 was the only remaining serotype whose genome sequence was not available. As a first step towards the molecular characterization of APMV-5, we have determined the growth characteristics and the complete genome sequence of APMV-5 strain Kunitachi. APMV-5 grew in many cell lines from different species, suggesting a broad *in vitro* host range for the virus. Interestingly APMV-5 grew in the amniotic cavity rather than the allantoic cavity of embryonated chicken eggs: the initial report describing the first isolation of APMV-5 had suggested that the virus did grow in the allantoic cavity of chicken eggs [24], but our findings are in agreement with a subsequent study reporting growth in the amniotic rather than allantoic cavity [23]. Also, APMV-5 strains have been reported to grow in the yolk sac of 6-day-old embryonated chicken eggs [42]. Infection of embryonated chicken eggs with 10^3^ Pfu/ml of APMV-5 via the amniotic sac did not cause embryo death even after 5 days post-infection, suggesting that the virus is avirulent in chickens.

Pairwise comparisons of the complete genome sequences of the prototype strains of the nine APMV serotypes showed that they shared 42 to 60% nt sequence identity (Table 1), and comparison of the amino acid sequences of cognate proteins of the various APMV serotypes also provided evidence of substantial divergence (Table 5 and data not shown). Thus, the serotype classification, which was originally based solely on the reactivity of antibodies that inhibit HA and NA activities, has now been substantiated by sequence analysis showing extensive divergence across the genome and involving all of the proteins.

The genome of APMV-5 strain Kunitachi is 17,262 nt in length, which is the longest genome of all of the APMVs analyzed to date (which have the following lengths: APMV-1, 15,186 nt; APMV-2, 14,904 nt; APMV-3, 16,272 nt; APMV- 4, 15,054 nt; APMV-6, 16,236 nt; APMV-7, 15,480 nt; APMV-8, 15,342 nt; and APMV-9, 15,438 nt). The APMV-5 genome also is longer than the average genome size (∼15,500 nt) of members of *Respirovirus*, *Morbillivirus*, *Rubulavirus* and *Avulavirus* sequenced to date, but is somewhat shorter than those of HeV (18,234 nt) and NiV (18,252 nt) of genus *Henipavirus* as well as those of some of the unclassified members of subfamily *Paramyxovirinae* such as Beilong virus, which has the longest paramyovirus genome (19,212 nt) identified to date [43]. The increased lengths of the genomes of HeV and NiV are mostly due to long downstream non-coding regions, whereas the increased genome length of the Beilong virus is due to an unusually large attachment protein, the presence of additional genes, and the presence of genome regions that may be either unusually long UTRs or may contain additional functional open reading frames. In the case of APMV-5, the increase in genome length compared to the other APMV serotypes was not due to additional genes or longer proteins, but rather was due to unusually long downstream UTRs in the P, M, and HN genes as well as a long trailer region. This suggested that, in the process of divergent evolution by the APMVs the change that was most readily accommodated was in the length of the downstream UTRs and the trailer region.

Apart from its longer length, the genome of APMV-5 had general similarities with the genomes of the other APMVs. The APMV-5 genome has six non overlapping genes (N, P, M, F, HN and L), similar to the genomes of all of the AMPVs except APMV-6, which has in addition the SH gene between the F and HN genes [19]. The lengths of the predicted APMV-5 proteins were very similar to those of the cognate proteins in other APMVs. Like the other APMVs, the genome length of APMV-5 is consistent with the ‘rule of six’, which appears to be a characteristic of all members of the subfamily *Paramyxovirinae* [3]. The leader sequence of APMV-5 is 55 nt long, a length that was exactly conserved among the other eight APMV serotypes and is generally conserved among members of the subfamily *Paramyxovirinae* [1]. The first 12–13 nt of the leader sequences in the subfamily *Paramyxovirinae* are more closely related within, than between genera, and this criteria is used in the classification of new isolates. Consistent with this, the first 12 nt of the APMV-5 leader sequence were found to be conserved among APMV -2, -6, -7, -8 and -9, suggesting a close evolutionary relationship among these viruses. The trailer region of APMV-5 is 552 nt in length, which is longer than the typical length (40–60 nt) for most members of the family *Paramyxoviridae* [44], and is the second longest trailer among APMVs, next to APMV-3 (707 nt) [17]. The first 12 nt of the 3′ leader sequence of APMV-5 are complementary to the last 12 nt sequence of the 5′ trailer regions suggesting the presence of conserved elements in the 3′ promoter regions of the genome and antigenome.

Comparison of the GS and GE sequences of APMV-5 with those of the other members of genus *Avulavirus* showed that the GE sequences are somewhat more highly conserved than the GS sequences. All of the GS sequences are rich in C residues (negative-sense), especially for the first five positions. However, only two positions were conserved among all nine serotypes, namely the C residues at positions 3 and 5. The GE sequences were rich in A and U (negative-sense): the first three positions of the signals of all nine serotypes were identical (AAU), and all of the sequences ended with a U tract that encodes the poly A tail. While all of the APMV genomes conform to the rule of six, there was no consistent pattern to the hexamer phasing positions of the GS and GE signals other than that of the N gene being at position 2 in all nine serotypes. Thus, while the importance of the hexamer phasing positioning is well-known for cis-acting signals involved in RNA replication [1], the importance of the phasing of transcription signals remains unclear. The IGS of APMV-5 differed from each other in sequence and varied in length from 4 to 57 nt. This variable, non-conserved nature of the IGS is shared with the other eight serotypes of APMV and also with the members of *Rubulavirus*, but differs from the conserved trinucleotide (usually GAA) IGS that is characteristic of *Respirovirus*, *Morbillivirus*, and *Henipavirus* [45]. With regard to the UTRs, the downstream UTRs (3′ in positive-sense) were longer than the upstream UTRs. This also is the case for the other APMVs (with the exception of the UTRs of the APMV-2 F gene and APMV-4 HN gene), and thus APMV-5 conforms to this general pattern. However, APMV-5 has unusually long 3′UTR in the P (458 nt), M (531 nt), and HN (378 nt) gene. Although the significance of UTR length in APMV-5 is not known, UTRs have been shown to modulate transcription and translation in APMV-1 [46], and measles [47] and canine distemper viruses [48].

APMV-5 strain Kunitachi also had other motifs in common with the other APMV serotypes and other members of the subfamily *Paramyxovirinae*. These include a conserved hydrophobic motif (**F**-X_4_-**Y**-X_4_-**SYAMG**) in the N protein that is thought to be involved in N-N self-assembly, a putative nuclear localization signal in the M protein, a putative “late domain” sequence in the M protein that may be involved in virion assembly, a putative sialic acid binding site in HN, proposed neuraminidase active-site residues in HN, and conserved polymerase and nucleotide-binding domains in the L protein. In addition, the editing motif and editing products of the APMV-5 P gene conform to those of the other APMV serotypes. The P gene of all members of the subfamily *Paramyxovirinae* is subjected to an RNA editing mechanism that causes a frame shift to access additional internal ORFs: the sole known exception is hPIV-1 of the genus *Respirovirus*. There are two patterns of editing within the subfamily. One pattern, which is followed by subfamily *Paramyxovirinae* except for genus *Rubulavirus*, involves expression of the P protein from the unedited mRNA and expression of the V protein from an mRNA that was edited to insert a single non-templated G residue. The insertion of two G residues results in an mRNA encoding the W, I, or D protein, depending on the virus. Editing by this group of viruses takes place at the editing motif 3′ UUUUUCCC 5′ (negative-sense), although this sequence varies somewhat in J virus and certain other unclassified viruses [1], [49]. Members of the *Rubulavirus* genus follow a second pattern in which the unedited mRNA encodes the V protein, and mRNA edited by the insertion of two G residues encodes the P protein [1]. The insertion of a single G residue results in expression of the I protein. The editing site and predicted protein products of the APMV-5 P gene are consistent with those of the other APMVs analyzed to date, and conform to the first pattern described above.

The F protein of the family *Paramyxoviridae* mediates fusion between the viral envelope and host plasma membrane at neutral pH. The F protein is synthesized as an inactive protein (F_0_) that is cleaved by host cell protease into the active form consisting of disulfide-linked F_2_−F_1_ subunits [1]. The sequence of the F protein cleavage site is a major determinant of NDV pathogenicity in chickens because it plays a major role in determining where and how the F_0_ precursor is cleaved [1], [50]. The cleavage sites of all known virulent strains of NDV have multiple basic residues (bold and underlined) that contain the preferred cleavage site of the intracellular cellular protease furin (**R**-X-**K/R**-**R**↓F) present in most cell types. In contrast, the cleavage site sequences of avirulent NDV strains characteristically have one or a few basic residues immediately upstream of the cleavage site that do not create a furin cleavage site. The ability of the NDV F protein to be cleaved by furin provides the opportunity for viral replication in a wide variety of tissues, which is essential for systemic spread of the virus. The presence of a non-furin cleavage site as in avirulent NDV strains restricts viral replication to the respiratory and enteric tracts, where secretory proteases necessary for F cleavage are present. The ability of NDV F to be cleaved also is affected by the amino acid that immediately follows the cleavage site and becomes the N-terminal residue of the F1 subunit: the presence of leucine in this position is associated with less efficient cleavage [34]. Surprisingly, however, some of the other APMV serotypes do not conform to the NDV paradigm. For example, for a number of the other eight serotypes, the sequence of the cleavage site does not predict whether exogenous protease is needed for growth *in vitro*. Specifically, the F protein cleavage sites of APMV-2 (PAS**R**↓F), APMV-4 (IQP**R**↓F) and APMV-7 (PSS**R**↓F) all contain a single basic residue (R) immediately upstream of the putative cleavage site, but do not require exogenous protease supplementation for growth in cell culture [16], [18], [20]. Other APMV serotypes do conform more closely with the NDV paradigm: specifically, they have a non-furin cleavage site and do require exogenous protease for replication in vitro. These viruses are APMV-3 (P**R**G**R**↓L), APMV-8 (PQT**R**↓L), and APMV-9 (**R**EG**R**↓I). It also is noteworthy that these three serotypes have leucine or isoleucine immediately downstream of the cleavage site. APMV-6 also has a single basic residue immediately upstream of the putative cleavage site (PEP**R**↓L), but the requirement for trypsin varies with the strain (S. Xiao, P.L.C. and S.K.S, unpublished data). APMV-5 strain Kunitachi contains five tandem basic amino acid residues at the putative cleavage site (^105^ **KRKKR**↓F^110^), and thus contains even more basic residues than most of the virulent strains of NDV sequenced to date. The presence of this highly basic cleavage sequence in APMV-5 may be responsible for the high pathogenicity of this virus in budgerigars, where it can cause up to 100% mortality [23]. However, it is interesting that this same virus was completely avirulent in chicken eggs. This is suggestive of a host range difference that is not ameliorated by the presence of a multi-basic cleavage site that otherwise would confer virulence in chickens. Similarly, some of the other APMV serotypes that did not require trypsin for cleavage *in vitro* nonetheless had low virulence in chickens (e.g. APMV-2, -4, and -7). Thus, for a number of the APMV serotypes, the cleavage site sequence did not predict the cleavage phenotype *in vitro* nor virulence *in vivo*.

All members of genus *Avulavirus* (APMV-1 to 9) have both hemagglutination and neuraminidase activities except for APMV-5 [23]. Although the APMV-5 HN protein has neuraminidase activity, it neither hemagglutinated nor hemadsorbed RBCs of chickens, turkeys, guinea pigs, horses, or human O-group, either at 37°C or at 4°C. The sequence of the APMV-5 stain Kunitachi HN protein provides evidence of the expected globular head, N-terminal-proximal signal/anchor domain, putative sialic acid binding motif (NRKSCS), seven conserved residues of the proposed neuraminidase active site, and eleven conserved cysteine residues that have been shown to be conserved among type II integral membrane proteins of subfamily *Paramyxovirinae* [38]. Thus, the lack of HA activity for APMV-5 cannot be ascribed to an obvious difference in HN structure. Non-hemagglutinating highly virulent APMV-1 strains which grow in the allantoic cavity, have been reported from cormorants (also a psittacine bird) in Canada [51]. These observations indicate that the HA activity of the HN protein is not required for replication of APMVs. APMV-5 failed to grow in the allantoic cavity of embryonated chicken eggs, in contrast to all hemagglutinating APMVs, which grow in the allantoic cavity. It is tempting to suggest that there is a link between these observations: specifically, that the lack of a strong HA in APMV-5 renders the virus unable to bind to receptors present on cells of the allantoic cavity. However, this hypothesis requires further study.

In summary, APMV-5 Kunitachi strain differs significantly from all other APMVs in the following ways; (i) having isolated only from budgerigars, (ii) the presence of an HN protein with no HA activity, (iii) the inability to grow in the allantoic cavity of embryonated chicken eggs, (iv) the presence of unusually long 3′UTRs in P, M and HN genes, and (v) the longest genome among the APMVs. The sequence alignment and phylogenetic analysis showed that APMV-5 clusters with the other APMVs, justifying its classification in the genus *Avulavirus*. Phylogenetically, APMV-5 is more closely related to APMV-6 than to the other known APMVs. Further sequence analysis of additional APMV-5 strains will help us understand the antigenic and genetic variation among APMV-5 strains. Future studies on the epidemiology and pathogenicity of this virus in different avian species will be necessary to define the role of this virus in avian health.

## References

[1] Lamb R, Parks G, Knipe DM, Howley PM, Griffin DE, Lamb RA, Martin MA, Roizman B, Straus SE (2007). *Paramyxoviridae*: the viruses and their replication..

[2] Lamb RA, Collins PL, Kolakofsky D, Melero JA, Nagai Y, Fauquet CM (2005). Family Paramyxoviridae..

[3] Calain P, Roux L (1993). The rule of six, a basic feature for efficient replication of Sendai virus defective interfering RNA.. J Virol.

[4] Kolakofsky D, Pelet T, Garcin D, Hausmann S, Curran J (1998). Paramyxovirus RNA synthesis and the requirement for hexamer genome length: the rule of six revisited.. J Virol.

[5] Curran J, Marq JB, Kolakofsky D (1995). An N-terminal domain of the Sendai paramyxovirus P protein acts as a chaperone for the NP protein during the nascent chain assembly step of genome replication.. J Virol.

[6] Peeples ME, Kingsbury DW (1991). Paramyxovirus M proteins : pulling it all together and taking it on the road.;.

[7] Lamb RA, Choppin PW (1977). The synthesis of Sendai virus polypeptides in infected cells. III. Phosphorylation of polypeptides.. Virology.

[8] Lin Y, Horvath F, Aligo JA, Wilson R, He B (2005). The role of simian virus 5 V protein on viral RNA synthesis.. Virology.

[9] Goodbourn S, Didcock L, Randall RE (2000). Interferons: cell signalling, immune modulation, antiviral response and virus countermeasures.. J Gen Virol.

[10] Alexander DJ, Saif YM (2003). Avian paramyxoviruses 2–9.;.

[11] Alexander DJ (1982). Avian paramyxoviruses-other than Newcastle disease virus.. World's Poul Sci J.

[12] Beck I, Gerlach H, Burkhardt E, Kaleta EF (2003). Investigation of several selected adjuvants regarding their efficacy and side effects for the production of a vaccine for parakeets to prevent a disease caused by a paramyxovirus type 3.. Vaccine.

[13] Alexander DJ, Collins MS (1982). Pathogenecity of PMV-3/Parakeet/Netherland/449/75 for chickens.. Avian Pathol.

[14] Jung A, Grund C, Muller I, Rautenschlein S (2009). Avian paramyxovirus serotype 3 infection in Neopsephotus, Cyanoramphus, and Neophema species.. J Avian Med Surg.

[15] Krishnamurthy S, Samal SK (1998). Nucleotide sequences of the trailer, nucleocapsid protein gene and intergenic regions of Newcastle disease virus strain Beaudette C and completion of the entire genome sequence.. J Gen Virol.

[16] Subbiah M, Xiao S, Collins PL, Samal SK (2008). Complete sequence of the genome of avian paramyxovirus type 2 (strain Yucaipa) and comparison with other paramyxoviruses.. Virus Res.

[17] Kumar S, Nayak B, Collins PL, Samal SK (2008). Complete genome sequence of avian paramyxovirus type 3 reveals an unusually long trailer region.. Virus Res.

[18] Nayak B, Kumar S, Collins PL, Samal SK (2008). Molecular characterization and complete genome sequence of avian paramyxovirus type 4 prototype strain duck/Hong Kong/D3/75.. Virology Journal.

[19] Chang PC, Hsieh ML, Shien JH, Graham DA, Lee MS (2001). Complete nucleotide sequence of avian paramyxovirus type 6 isolated from ducks.. J Gen Virol.

[20] Xiao S, Paldurai A, Nayak B, Subbiah M, Collins PL (2009). Complete genome sequence of avian paramyxovirus type 7 (strain Tennessee) and comparison with other paramyxoviruses.. Virus Res.

[21] Paldurai A, Subbiah M, Kumar S, Collins PL, Samal SK (2009). Complete genome sequences of avian paramyxovirus type 8 strains goose/Delaware/1053/76 and pintail/Wakuya/20/78.. Virus Res.

[22] Samuel AS, Kumar S, Madhuri S, Collins PL, Samal SK (2009). Complete sequence of the genome of avian paramyxovirus type 9 and comparison with other paramyxoviruses.. Virus Res.

[23] Nerome K, Nakayama M, Ishida M, Fukumi H (1978). Isolation of a new avian paramyxovirus from budgerigar (Melopsittacus undulatus).. J Gen Virol.

[24] Mustaffa-Babjee A, Spradbrow PB, Samuel JL (1974). A pathogenic paramyxovirus from a budgerigar (Melopsittacus undulatus).. Avian Dis.

[25] Gough RE, Manvell RJ, Drury SE, Naylor PF, Spackman D (1993). Deaths in budgerigars associated with a paramyxovirus-like agent.. Vet Rec.

[26] Kumar S, Tamura K, Nei M (2004). MEGA3: Integrated software for Molecular Evolutionary Genetics Analysis and sequence alignment.. Brief Bioinform.

[27] Samal SK, Collins PL (1996). RNA replication by a respiratory syncytial virus RNA analog does not obey the rule of six and retains a nonviral trinucleotide extension at the leader end.. J Virol.

[28] Miller PJ, Boyle DB, Eaton BT, Wang LF (2003). Full-length genome sequence of Mossman virus, a novel paramyxovirus isolated from rodents in Australia.. Virology.

[29] Yu M, Hansson E, Shiell B, Michalski W, Eaton BT (1998). Sequence analysis of the Hendra virus nucleoprotein gene: comparison with other members of the subfamily Paramyxovirinae.. J Gen Virol.

[30] Coleman NA, Peeples ME (1993). The matrix protein of Newcastle disease virus localizes to the nucleus via a bipartite nuclear localization signal.. Virology.

[31] Schmitt AP, Leser GP, Morita E, Sundquist WI, Lamb RA (2005). Evidence for a new viral late-domain core sequence, FPIV, necessary for budding of a paramyxovirus.. J Virol.

[32] Hosaka M, Nagahama M, Kim WS, Watanabe T, Hatsuzawa K (1991). Arg-X-Lys/Arg-Arg motif as a signal for precursor cleavage catalyzed by furin within the constitutive secretory pathway.. J Biol Chem.

[33] Servan de Almeida R, Maminiaina OF, Gil P, Hammoumi S, Molia S (2009). Africa, a reservoir of new virulent strains of Newcastle disease virus?. Vaccine.

[34] Morrison TG (2003). Structure and function of a paramyxovirus fusion protein.. Biochim Biophys Acta.

[35] Varghese JN, Laver WG, Colman PM (1983). Structure of the influenza virus glycoprotein antigen neuraminidase at 2.9 A resolution.. Nature.

[36] Mirza AM, Deng R, Iorio RM (1994). Site-directed mutagenesis of a conserved hexapeptide in the paramyxovirus hemagglutinin-neuraminidase glycoprotein: effects on antigenic structure and function.. J Virol.

[37] Takimoto T, Taylor GL, Crennell SJ, Scroggs RA, Portner A (2000). Crystallization of Newcastle disease virus hemagglutinin-neuraminidase glycoprotein.. Virology.

[38] Langedijk JP, Daus FJ, van Oirschot JT (1997). Sequence and structure alignment of Paramyxoviridae attachment proteins and discovery of enzymatic activity for a morbillivirus hemagglutinin.. J Virol.

[39] Poch O, Blumberg BM, Bougueleret L, Tordo N (1990). Sequence comparison of five polymerases (L proteins) of unsegmented negative-strand RNA viruses: theoretical assignment of functional domains.. J Gen Virol.

[40] Schnell MJ, Conzelmann KK (1995). Polymerase activity of in vitro mutated rabies virus L protein.. Virology.

[41] Malur AG, Choudhary SK, De BP, Banerjee AK (2002). Role of a highly conserved NH(2)-terminal domain of the human parainfluenza virus type 3 RNA polymerase.. J Virol.

[42] Purchase HG (January 1990). A Laboratory Manual for the Isolation and Identification of Avian Pathogens Kendall/Hunt Publishing Company..

[43] Li Z, Yu M, Zhang H, Magoffin DE, Jack PJ (2006). Beilong virus, a novel paramyxovirus with the largest genome of non-segmented negative-stranded RNA viruses.. Virology.

[44] Shioda T, Iwasaki K, Shibuta H (1986). Determination of the complete nucleotide sequence of the Sendai virus genome RNA and the predicted amino acid sequences of the F, HN and L proteins.. Nucleic Acids Res.

[45] Nylund S, Karlsen M, Nylund A (2007). The complete genome sequence of the Atlantic salmon paramyxovirus (ASPV).. Virology.

[46] Yan Y, Rout SN, Kim SH, Samal SK (2009). Role of untranslated regions of the hemagglutinin-neuraminidase gene in replication and pathogenicity of newcastle disease virus.. J Virol.

[47] Takeda M, Ohno S, Seki F, Nakatsu Y, Tahara M (2005). Long untranslated regions of the measles virus M and F genes control virus replication and cytopathogenicity.. J Virol.

[48] Anderson DE, von Messling V (2008). Region between the canine distemper virus M and F genes modulates virulence by controlling fusion protein expression.. J Virol.

[49] Jack PJ, Boyle DB, Eaton BT, Wang LF (2005). The complete genome sequence of J virus reveals a unique genome structure in the family Paramyxoviridae.. J Virol.

[50] Panda A, Huang Z, Elankumaran S, Rockemann DD, Samal SK (2004). Role of fusion protein cleavage site in the virulence of Newcastle disease virus.. Microb Pathog.

[51] Weingartl HM, Riva J, Kumthekar P (2003). Molecular characterization of avian paramyxovirus 1 isolates collected from cormorants in Canada from 1995 to 2000.. J Clin Microbiol.

